# Implementation Strategies for Web-Based Apps for Screening: Scoping Review

**DOI:** 10.2196/15591

**Published:** 2020-07-20

**Authors:** Chor Yau Ooi, Chirk Jenn Ng, Anne E Sales, Hooi Min Lim

**Affiliations:** 1 University of Malaya eHealth Initiative, Department of Primary Care Medicine Faculty of Medicine University of Malaya Kuala Lumpur Malaysia; 2 Department of Family Medicine Faculty of Medicine and Health Sciences Universiti Malaysia Sarawak Kota Samarahan Malaysia; 3 Center for Clinical Management Research Veterans Affairs Ann Arbor Healthcare System Ann Arbor, MI United States; 4 University of Michigan Medical School Ann Arbor, MI United States

**Keywords:** internet, mHealth, eHealth, mass screening, implementation strategies

## Abstract

**Background:**

Screening is an effective primary prevention strategy in health care, as it enables the early detection of diseases. However, the uptake of such screening remains low. Different delivery methods for screening have been developed and found to be effective in increasing the uptake of screening, including the use of web-based apps. Studies have shown that web-based apps for screening are effective in increasing the uptake of health screening among the general population. However, not much is known about the effective implementation of such web-based apps in the real-world setting. Implementation strategies are theory-based methods or techniques used to enhance the adoption, implementation, and sustainability of evidence-based interventions. Implementation strategies are important, as they allow us to understand how to implement an evidence-based intervention. Therefore, a scoping review to identify the various implementation strategies for web-based apps for screening is warranted.

**Objective:**

This scoping review aims to identify (1) strategies used to implement web-based apps for health screening, (2) frameworks used for implementing web-based apps for health screening, (3) outcome measures of implementation strategies, and (4) effective implementation strategies.

**Methods:**

This scoping review was conducted based on Arksey and O’Malley’s framework. After identifying the review question, two researchers independently screened and selected relevant literature from PubMed, Embase, Cochrane, Cumulative Index of Nursing and Allied Health Literature, PsycINFO, International Standard Randomised Controlled Trial Number Registry, OpenGrey, ClinicalTrials.gov, World Health Organization International Clinical Trials Registry Platform, and Web of Science. This was followed by charting the data using a standardized form. Finally, we collated, summarized, and reported the results quantitatively and qualitatively based on the review objectives.

**Results:**

A total of 16,476 studies were retrieved, of which 5669 were duplicates. From a total of 10,807 studies, 10,784 studies were excluded based on their titles and abstracts. There were 23 full-text articles reviewed, and 4 articles were included in the final analysis. Many studies were excluded because they focused on the effectiveness and not on the implementation of web-based apps. Facilitation was the most cited implementation strategy used, followed by reminders, clinical champions, and educational meetings and materials. Only 2 studies used implementation frameworks to guide the evaluation of their studies. Common outcome measures for implementation strategies were feasibility, fidelity, and penetration. Implementation strategies reported to be effective were quality improvement meetings, facilitation, educational meetings, and clinical champions.

**Conclusions:**

There is a dearth of literature on the implementation of web-based apps for health screening. Implementation strategies were developed without any reported use of implementation theories or frameworks in most studies. More research on the development and evaluation of web-based screening app implementations is needed.

## Introduction

### Background

Screening is one strategy for health promotion and disease prevention. Screening is the process of identifying healthy people who may be at an increased risk of a particular disease or health problem [1]. Screening enables early detection of disease so that steps can be taken quickly, when relatively little damage has been done, to prevent the disease from progressing [2].

### Prior Research

Many studies have shown screening to be effective in the prevention of diseases. A study in Korea reported that participation in cardiovascular disease (CVD) health screening was associated with lower CVD and all-cause mortality from CVD. There was also an increase in the early detection of CVD and a reduction in health care use and cost [3]. A systematic review of colorectal cancer (CRC) screening using fecal occult blood test showed that it is effective in reducing CRC mortality [4]. However, not all health screening tests are beneficial [5]. Studies have shown, for example, that prostate-specific antigen testing has led to overdiagnosis in up to 40% of the patients screened [6]. Screening tests are not uniformly accurate and are liable to false-positive results and overdiagnosis, which can cause harm to patients [5]. Therefore, care is needed in discussing the pros and cons of screening tests with patients, and to come to shared decision making about screening [5].

### Methods of Screening Delivery

However, even when screening is beneficial and effective, its uptake remains suboptimal [7]. One reason is that screening cannot be done using just a single method, a “one size fit all” solution [7]. Different delivery methods must be used to address patient needs. A systematic review looking at improving health screening uptake in men showed that there was a variety of delivery methods for screening, ranging from use of education materials or video decision aids to web-based approaches [8].

Information and communication technology (ICT) has become an important platform to improve health care in the general population [9,10]. ICT use in health is sometimes also known as electronic health (eHealth) [11]. Many forms of eHealth interventions have been developed to improve public health care, in particular, web-based apps [12]; although, the use of electronic health records is another approach using ICT. Our focus in this study was on web-based approaches that do not require the use of an electronic health record, which is often not available, especially in low-resource settings.

A web-based app is defined as a program that is accessed over a network connection using HTTP rather than existing within a device’s memory. Web-based apps often run inside a web browser. However, web-based apps may also be client-based, where a small part of the program is downloaded to a user’s desktop, but processing is done over the internet on an external server [13]. With the proliferation of the internet and the accessibility of Wi-Fi and mobile data, web-based apps have become highly accessible to the general population, even in low-resource settings. The availability of smartphones enables the general population to have easy access to many web-based apps instantaneously from anywhere around the world. Web-based apps addressing different health needs like health screening, health promotion, and health advice have proliferated over the last decade. Many studies have shown that these web-based apps can be effective in changing health behaviors and improving health status [12]. For example, a completed systematic review showed that computer-based interventions are effective in improving sexual health knowledge [14].

Web-based apps for screening use questionnaires to accomplish screening. This screening can include mental and behavioral health as well as risk factor assessment. Examples include screening for depression risk, alcoholic addiction, and smoking. Users (patients) enter their responses based on the questions, and the web app sometimes provides appropriate advice to the patient. Several studies have shown that web-based apps for screening are effective in increasing the uptake of health screening among the population [15-17]. However, how successful implementation of these web-based health apps is accomplished has not yet been studied.

### Rationale for Implementation Research

Implementation research involves the study of “the use of strategies to adopt and integrate evidence-based health interventions and change practice patterns within specific settings” [18]. Implementation science approaches use a systematic process of getting an evidence-based intervention (EBI) to reach the target population. Many frameworks, theories, and models are available to guide the implementation process [19]. By using frameworks appropriately to guide the implementation process, including development and design of strategies, *implementation strategies* can be developed to facilitate the uptake of EBIs in a specific health care setting. *Implementation strategies* are activities to enhance the adoption, implementation, and sustainability of EBIs [20]. Explicit implementation increases the likelihood of getting the EBI to the target population compared to leaving it to usual processes. For example, a study done in a clinic found that by attaching a reminder to each patient’s chart about guidelines on the management of dyslipidemia, 94% of patients received the recommended treatment compared to just 35% in the control group [21].

Nonetheless, studies have shown that many EBIs do not reach their target population [22]. There is an obvious evidence-practice gap. Considerable effort is expended on the discovery of new EBIs but not on examining how these EBIs can be successfully implemented in health care settings [18]. A previous scoping review showed that limited research has been done to implement internet interventions for depression [23]. Therefore, there is a need to increase the amount of implementation to ensure that this evidence-practice gap can be reduced.

Studies show that implementing ICT in health care settings can be complex [23-25]. Systematic reviews demonstrate that organizational factors, setting, integration of the EBI into the workflow, and contextual and societal factors are important influences on the success of implementation [23,24]. However, most studies have focused more on the features of the ICT app and characteristics of end-users than on implementation factors or determinants [24]. Studies of the process of implementation and the factors associated with it are also lacking in the context of ICT in health care [24]. A systematic review demonstrated that studying the implementation process and factors influencing it are important in determining the success of implementation [23]. It is important to evaluate barriers and facilitators to successful implementation of an EBI [24].

Studying *implementation strategies* allows us to understand how to implement a particular EBI [26]. As noted in the literature, developing implementation strategies to overcome barriers to implementation is an important research agenda. Implementation strategies can be critical in bridging the evidence-practice gap when appropriately designed and deployed. Recognizing the importance of implementation strategies, various studies have been done to identify effective implementation strategies for various types of EBI [27]. However, there is a scant of literature on implementation strategies for web-based screening apps. A search of the PROSPERO (International Prospective Register of Systematic Reviews) database of systematic review protocols found no systematic reviews in this area. Therefore, a scoping review to identify the various implementation strategies for web-based screening apps appeared appropriate.

## Methods

### Overview

This scoping review uses the framework by Arksey and O’Malley [28] and Levac et al [29]. This methodology was selected because our aim is to explore the variation in implementation strategies used to implement web-based health screening apps. The Arksey and O’Malley framework is comprised of six stages: (1) identifying the research question; (2) identifying relevant studies; (3) study selection; (4) charting the data; (5) collating, summarizing, and reporting the results; and (6) consultation exercise.

### Stage 1: Identifying the Research Question

Among studies exploring the implementation of web-based screening apps:

1. What implementation strategies were used and proven effective?
2. What studies were informed by implementation theories, models, and frameworks, and which ones in particular?
3. What implementation outcomes were measured?
4. What factors were identified as influencing implementation effectiveness?

### Stage 2: Identifying Relevant Studies

The literature search was conducted using PubMed, Embase, Cochrane, CINAHL (Cumulated Index to Nursing and Allied Health Literature) via EBSCOHost, PsycINFO via OvidSP, ISRCTN (International Standard Randomised Controlled Trials Number) registry, OpenGrey, ClinicalTrials.gov, World Health Organization International Clinical Trials Registry Platform, and Web of Science for peer-reviewed scientific literature. The search was conducted in August 2018 for 2 weeks and concluded on August 28, 2018. Other search methods used to supplement the literature include reference or footnote tracking, using the “related articles” function in PubMed, citation tracking, personal knowledge and personal contacts, and contacting experts in the field. The search terms and search strategies are included in Multimedia Appendix 1.

### Stage 3: Study Selection

The inclusion criteria were studies that described any implementation strategies used to implement web-based apps for screening. Definition of specific terms are outlined as follows:

- Implementation strategies: methods or techniques used to enhance the adoption, implementation, and sustainability of EBIs [20]
- Web-based app: any program accessed over a network connection using HTTP rather than existing within a device’s memory [13]
- Frameworks: A framework is a structure, overview, outline, system, or plan consisting of various descriptive categories (eg, concepts, constructs, or variables) and the relations between them that are presumed to account for a phenomenon [19].

The study must also include all age groups and genders; quantitative, qualitative, and mixed-method study designs will be included, and studies must be in the English language.

The exclusion criteria were as follows:

1. Non–web-based apps such as desktop-based, computer-based, CD-ROM interventions, mobile apps, and electronic health records
2. Nonempirical references such as trial protocols, book reviews, editorials, magazine articles, and theoretical or methodological articles
3. Non-English studies

Two independent reviewers screened the titles and the abstracts. The full texts of relevant articles were retrieved and screened by the two independent reviewers. Any disagreements were discussed among the two reviewers, and a third reviewer was consulted if the two reviewers were unable to resolve any disagreements.

### Stage 4: Charting the Data

Data were extracted using a standardized form. Two reviewers independently extracted the data. Any disagreements were resolved with a third reviewer. Data extracted from individual studies include author(s), year of publication, origin or country of origin (where the study was published or conducted), aims or purpose, frameworks, study population and sample size (if applicable), methodology or methods, intervention type, comparator and details of these (eg, duration of the intervention; if applicable), duration of the intervention (if applicable), outcomes and details of these (eg, measures; if applicable), and how the key findings relate to the scoping review question(s). The two reviewers met after extracting data from two articles to determine if the data extraction process was consistent with the research questions and purpose. Any inconsistencies were discussed until a consensus was reached. A third researcher was consulted when the two researchers were unable to reach a consensus.

### Stage 5: Collating, Summarizing, and Reporting the Results

The results of this review were divided into three distinct steps based on recommendations by Levac et al [29]: (1) analysis (descriptive numerical summary analysis and qualitative thematic analysis); (2) reporting the results according to the research questions; and (3) considering the meaning of the findings as they relate to the overall study purpose and discuss implications for future research, practice, and policy.

### Stage 6: Consultation Exercise

The consultation exercise was conducted among experts in primary care, eHealth, and implementation science. The experts discussed the selection of articles and helped troubleshoot the issues that occurred during the review process. This exercise helped to validate and shape the study outcomes of this review.

### Quality Assessment

Quality assessment of the studies was done using the Mixed Methods Appraisal Tool (MMAT). MMAT was used as it is a validated tool to quickly assess qualitative, quantitative, and mixed methods studies [30]. This was done to assess the quality of the studies and not used as criteria for the inclusion of studies. The table reporting the quality assessment is included in Multimedia Appendix 2.

## Results

### Included Studies

**Figure 1:**
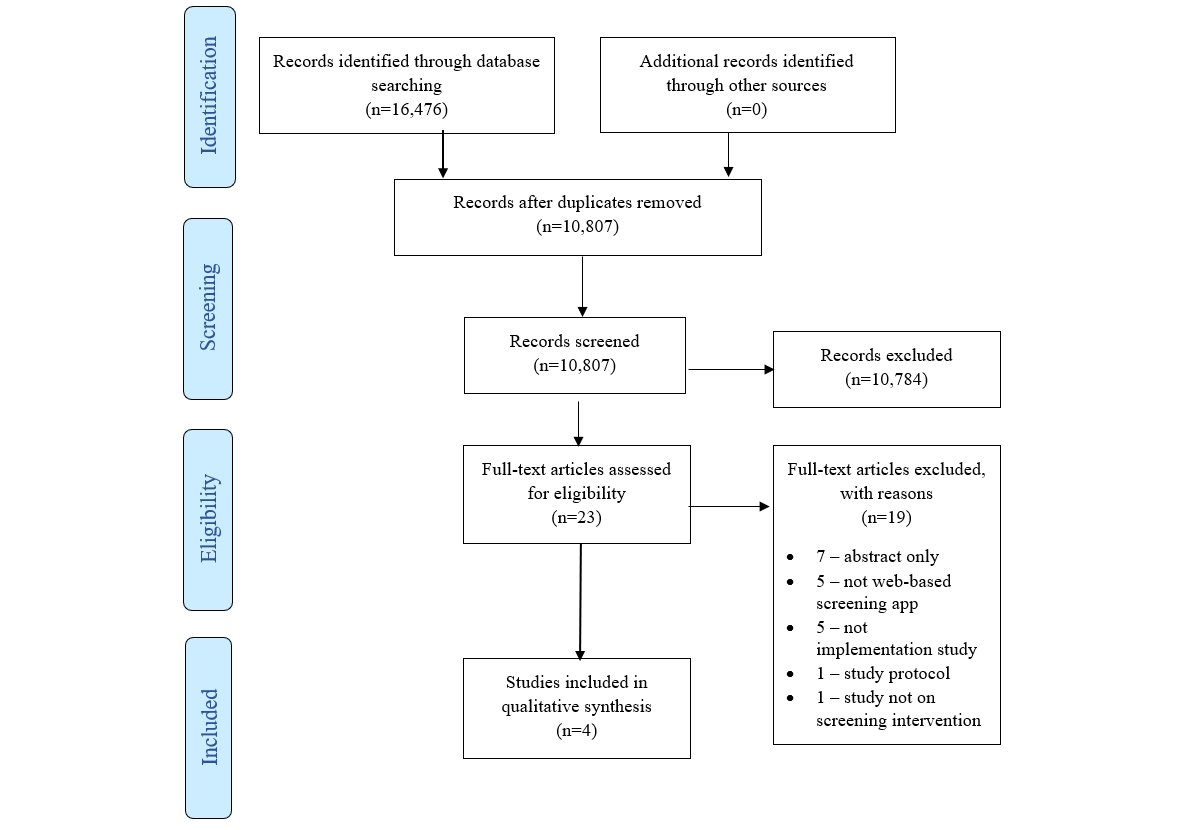
Flow diagram of selection of studies.

The searching databases yielded a total of 16,476 records. After the initial screening and removal of duplicates, 4 studies were included in this review (Figure 1).

Most studies were excluded at the title or abstract screening phase because the aim of this review was to identify implementation strategies for web-based apps. Most studies found through the database search evaluated the effectiveness of the web-based apps rather than focusing on their implementation. Only 4 studies fulfilled the inclusion criteria [31-34]. Of these studies, 3 were conducted in primary care settings and 1 in an emergency department. All the studies were descriptive in nature, and the study duration ranged from 2 to 15 months. The characteristics of each study are summarized in Table 1.

**Table 1:** Characteristics of included studies.

Authors	Year	Intervention	Setting	Study population	Methods	Implementation duration
Webb et al [31]	2018	Health and lifestyle screening tool (“app”), Check Up GP^a^ for young people in an Australian general practice	Australia General practice	Patients: young people 14-25 years of age HCP^b^: GP, practice manager, reception coordinator, and receptionists	Qualitative: semistructured interviews and focus groups Quantitative: cross-sectional survey	2 months
Diez-Canseco et al [32]	2018	A web-based mental health screening app	Peru Primary care	Patients: adults ≥18 years of age HCP: midwives, nurses, and nurse assistants	Mixed methods Qualitative: face-to-face structured interviews with HCPs and patients Quantitative: web-based data collection platform	9 weeks
Krist et al [33]	2014	MOHR^c^, a web-based health risk assessment tool	United States Primary care	Patients: adults HCP: clinicians, front desk staff, practice rooming staff, and medical assistants	Cluster-randomized, mixed methods implementation trial Qualitative: interviews with HCPs Quantitative: data from research networks and the MOHR tool	10 months
Scribano et al [34]	2011	Computerized intimate partner violence screening	United States Hospital emergency department	Patients: adults HCP: emergency department staffs	Qualitative: direct observation of patient use, feedback from patients, one-on-one feedback from emergency department staff, and team meetings Quantitative: questionnaire survey through kiosk.	15 months

^a^GP: general practitioner.

^b^HCP: health care provider.

^c^MOHR: My Own Health Report.

### Quality Assessment

The single randomized controlled trial included in this review was of moderate quality [33]. The 2 arms were not comparable at baseline, and the outcome assessors were not blinded [33]. The other 3 studies contained both qualitative and quantitative components [31,32,34]. For the qualitative component, 2 studies were of good quality [31,32]. As there was no clear descriptions on how the data were collected, analyzed, and interpreted, 1 study was considered to be of poor quality [34]. For the quantitative component, 2 studies were of moderate quality, as the sample population and the risk of nonresponse bias of the studies were not clearly described [32,34]. The other study was of good quality [31].

### Implementation Frameworks and Strategies for Web-Based Screening Apps

Only 2 studies used frameworks to guide and evaluate the process and outcomes of the study. However, in both studies, a framework and theory were used to guide the evaluation of the studies and not on the development of the implementation strategies. Table 2 summarizes the implementation strategies and frameworks used in each study. The types of frameworks were categorized based on Nilsen’s [19] taxonomy of implementation frameworks. The implementation strategies are categorized based on the Effective Practice and Organisation of Care taxonomy [35].

**Table 2:** Implementation frameworks and strategies used for web-based apps.

Authors	Frameworks	Types of framework [19]	Implementation strategies [35]	Implementation activities
Webb et al [31]	NPT^a^	Implementation theories		
			Reminder	Phone call and SMS as reminder to patients to complete the app
			Reminder and facilitation	Receptionist to prompt patient to complete app in the waiting room using tablet
			Continuous quality improvement	Quality improvement meetings with HCPs^b^
			Educational materials	Provision of educational documents to support HCPs
Diez-Canseco et al [32]	None	N/A^c^		
			Leadership engagement/buy-in	Engaging policy makers for support and buy-in
			Educational meetings	Training for HCPs
			Technical assistance	Telephone and face-to-face support, and supervision for HCPs throughout the implementation period
			Coaching	Regular supervision meetings with HCPs to troubleshoot problems encountered throughout the implementation period
Krist et al [33]	RE-AIM^d^	Evaluation framework		
			Clinical champions	Appointment of practice champions
			Educational meetings	Training for HCPs
			Facilitation	Mailed invitations to complete the app
			Facilitation	Kiosk provided in the clinic waiting room to complete the app with help from researcher
			Facilitation	Completion of the app via phone call by researcher
			Facilitation	Completion of the app via tablet with help from either researcher, practice rooming staff, or medical assistant in the clinic waiting room
Scribano et al [34]	None	N/A		
			Patient education and facilitation	Nurses and receptionists provided instruction forms to patients for the screening kiosks
			Environment	Placing screening kiosks in strategic locations
			Clinical champions	Appointment of practice champions

^a^NPT: Normalization Process Theory.

^b^HCP: health care provider.

^c^N/A: not applicable.

^d^RE-AIM: Reach, Effectiveness, Adoption, Implementation, Maintenance.

Facilitation was the most cited strategy used. Other strategies that were cited more than once included reminders, clinical champions, and educational meetings.

### Outcome Measures

The implementation outcomes for the studies included in this review were categorized based on the taxonomy of implementation outcomes by Proctor et al [36]. The implementation outcomes can be broadly categorized into acceptability, adoption, appropriateness, feasibility, fidelity, implementation cost, penetration, and sustainability. Table 3 outlines the outcome measures reported in the studies.

**Table 3:** Outcome measures.

Authors, Outcomes measured		Implementation outcome
**Webb et al [31]**		
	Number of times needed to provide support to staff on the use of the app	Feasibility
	Location completed Check Up GP^a^	Feasibility
	If patients received an SMS with a link to Check Up GP before attending the practice	Fidelity
	If patients felt that they had sufficient privacy when completing the app	Fidelity
	Postimplementation staff interviews and focus group discussions based on NPT^b^	Acceptability, adoption, appropriateness, feasibility, fidelity, sustainability
**Diez-Canseco et al [32]**		
	Number of screenings	Penetration
	Fidelity to screening	Fidelity
	Integration of screening into routine clinical service	Sustainability
**Krist et al [33]**		
	Reach: proportion of eligible patients approached who completed a MOHR^c^ assessment	Penetration
	Adoption: percentage of practices approached for study participation who agreed to participate	Adoption
	Implementation: how practices integrated MOHR into their workflow and the time and staff needed to carry out implementation steps	Fidelity
	Maintenance: whether early intervention practices continued to use MOHR after the study	Sustainability
**Scribano et al [34]**		
	Number of screenings completed by patients	Penetration
	Computer technology failure rate	Feasibility

^a^GP: general practitioner.

^b^NPT: Normalization Process Theory.

^c^MOHR: My Own Health Report.

### Effective Implementation Strategies

Different studies revealed different implementation strategies that were reported to be effective in their settings (Textbox 1).

For Webb et al [31], the quality improvement meetings using the Plan-Do-Act-Study framework were reported to be effective. The study also reported that facilitation by researchers on-site helped in the implementation process. Comparing different sites using the web-based app, the authors found that using the web-based app in the waiting room was the most effective compared to other settings (84%) [31].

**Effective implementation strategies.**

**Health care providers**

- Quality improvement meetings
- Facilitation
- Educational meetings
- Clinical champions

**Patients**

- Environment
- Facilitation

In the study conducted by Diez-Canseco et al [32], the authors stated that training health care providers was the key to promote use of the web-based app. Supervision was another important strategy to troubleshoot issues when implementing the web-based app.

For Krist et al [33], the authors compared implementation strategies used in nine different primary care clinics. They found that the strategy with the highest screening completed was to complete the app in the clinic waiting room with facilitation from the researcher (94.4% completion rate). Overall, they found that facilitation from staff in the clinic to help the patient complete the web-based app was the key to successful implementation. There was a higher completion rate with facilitation from staff compared to no facilitation (71.2% vs 30.3%) [33].

In the study by Scribano et al [34], appointing nurse champions helped to increase the screening rate. Another strategy that improved the screening rate was moving the kiosk to a more strategic location in the emergency department.

## Discussion

### Principal Findings

This review revealed that there is a very small set of literature reporting on the implementation of web-based apps for screening. Many studies evaluated the effectiveness or application of web-based screening apps, but very few described how to implement them [10,25]. The lack of literature found in this review suggests that there is a need for more implementation studies to be conducted in this area. Many studies have shown that implementation will not happen on its own, and implementation has to be planned to succeed [37].

Integrating an implementation study with an effectiveness study may be one way forward. Hybrid study designs may yield more information, as both the effectiveness of the intervention and the implementation can be assessed in a single study [38]. This may also reduce the time needed to translate an EBI into practice. However, these study designs require more expertise, resources, and manpower to conduct [38]. Therefore, it may not be feasible to conduct a hybrid study in settings with limited resources.

In this review, the most common implementation strategies used in implementing web-based apps were facilitation, education, and clinical champions. Most of these strategies targeted only patients and health care providers. Only 1 study mentioned strategies related to leadership engagement [32]. The strategies found in this review were limited to smaller scales and local contexts.

There was also no clear discussion on how strategies were developed or designed for implementation. Using a systematic process and determinant frameworks to help guide the development of strategies can be important for successful implementation and evaluation [19]. Having a systematic process for developing strategies will also help others replicate the process if the implementation was successful. We found that only 2 of the 4 studies used implementation frameworks or theories. This is consistent with the implementation research literature [19]. Without a solid theoretical foundation, it is challenging to explain why an implementation fails or succeeds [19]. Currently, many theories and frameworks are available for implementation research [19]. By using a combination of these theories or frameworks, researchers may better understand how to plan an implementation study systematically. By testing strategies and frameworks, new knowledge can be gained for future implementation studies.

In this review, different outcome measures were used in each study. However, there was no clear explanation of how the authors of the studies determined which outcome to measure. Which implementation strategies contributed to the outcomes were not clear because several different strategies were used in most studies, and their individual effects could not be evaluated. Therefore, we were unable to conclude which implementation strategies were useful. The lack of implementation frameworks or theories used in the studies may have contributed to this issue. The process of developing implementation strategies should be done systematically, ideally to link each strategy to different outcomes. If this is feasible, it could allow us to assess which strategies work. This may also allow researchers to further explore why a particular strategy works for a specific outcome. Lack of effect may lie with the implementation strategy itself or may be related to how the implementation strategy was implemented. Therefore, it is crucial that researchers give adequate thought to the implementation outcomes they measure and how an implementation strategy might affect each outcome. In addition, the field of implementation research is shifting from an exclusive focus on the effectiveness of implementation strategies to a focus on how strategies achieve their effect, if any, and on the factors that make implementation, across a spectrum of evidence-based practices, including ICT approaches, either more or less likely to be successful. Future studies will hopefully include this broader focus, as the current literature does not go beyond a simplistic examination of the implementation strategies’ effectiveness.

### Strengths and Limitations

This review may be limited because only English language studies were included. Another limitation could have been related to screening titles and abstracts. This may have limited the inclusion of studies if implementation strategies were not discussed in the abstract. However, based on our review, we found that most of the implementation strategies could be identified from abstracts alone. To this end, this scoping review revealed that only a few studies addressed the implementation of web-based health screening apps, and therefore, more research is needed in this area.

### Conclusion

This scoping review shows that the study of implementation of web-based apps for screening is still in its infancy. Many studies have assessed the effectiveness of web-based apps, but only a few have focused on how to implement them. We were able to identify different implementation strategies for implementing web-based apps for screening. However, there is little evidence that the strategies reported were systematically developed using theories or frameworks. The lack of frameworks and theories used in these studies was also evident. There is a need to study not only the effectiveness of implementation strategies but the process of implementation and how this affects outcomes. This review shows that more work is still needed to study implementation of web-based apps for screening in a systematic process based on implementation theories and frameworks.

## Appendix

Multimedia Appendix 1 Search terms and search strategy for scoping review.

Multimedia Appendix 2 Mixed Method Appraisal Tool Table.

## Abbreviations

CINAHL: Cumulated Index to Nursing and Allied Health Literature
CRC: colorectal cancer
CVD: cardiovascular disease
EBI: evidence-based intervention
eHealth: electronic health
ICT: information and communication technology
ISRCTN: International Standard Randomised Controlled Trials Number
MMAT: Mixed Methods Appraisal Tool
PROSPERO: International Prospective Register of Systematic Reviews
