# Supplementary material for: Implementation Strategies for Web-Based Apps for Screening: Scoping Review
Source: J Med Internet Res. 2020 Jul 20;22(7):e15591. doi: 10.2196/15591 (PMC7400029; doi:10.2196/15591)
Supplement: Multimedia Appendix 1 [file jmir_v22i7e15591_app1.docx]

**Search terms and search strategy for Scoping Review**

1. PubMed search strategy

| No. | Search strategy |
| --- | --- |
| #5 | (#1 AND #2 AND #3 NOT #4) |
| #4 | review[pt] OR letter[pt] OR editorial[pt] OR news[pt] OR newspaper article[pt] OR patient education handout[pt] OR case reports[pt] OR historical article[pt] |
| #3 | Screening[tiab] OR Early detection of cancer[mh] OR Mass screening[mh] OR Health check*[tiab] OR Health examination[tiab] OR health promotion[tiab] OR occult blood[tiab] OR sigmoidoscopy[tiab] OR colonoscopy[tiab] OR cervical smear[tiab] OR cervical screen[tiab] OR pap smear[tiab] OR pap test[tiab] OR papanicolaou smear[tiab] OR papanicolaou test[tiab] OR mammography[tiab] OR prostate-specific antigen[tiab] |
| #2 | Web[tiab] OR Internet[tiab] OR Internet[mh] OR Net surfing[tiab] OR technology-based[tiab] OR Information Technology[tiab] OR Website[tiab] OR information and communication technology[tiab] OR eHealth[tiab] OR telemedicine[tiab] OR telemedicine[mh] OR ICT[tiab] OR Telehealth[tiab] OR email[tiab] |
| #1 | Implement*[tiab] OR Intervention[tiab] OR Adoption[tiab] OR Integration[tiab] OR Feasibility[tiab] OR Health plan implementation[mh] OR Delivery of health care[mh] OR Knowledge transfer[tiab] OR Diffusion of innovation[mh] OR Diffusion[tiab] OR Disseminat*[tiab] OR Translat*[tiab] OR Quality[tiab] OR Program[tiab] OR Innovation[tiab] |

1. CINAHL via EBSCOhost

| No. | Search strategy |
| --- | --- |
| #5 | (#1 AND #2 AND #3 NOT #4) |
| #4 | PT “Review” OR PT “letter” OR PT “editorial” OR PT “historical material” |
| #3 | TI “Screening” OR AB “screening” OR MH “health screening” OR MH “cancer screening” OR TI “Health check*” OR AB “health check*” OR TI “Health examination” OR AB “health examination” OR TI “health promotion” OR AB “health promotion” OR TI “occult blood” OR AB “occult blood” OR TI “sigmoidoscopy” OR AB “sigmoidoscopy” OR TI “colonoscopy” OR AB “colonoscopy” OR TI “cervical smear” OR AB “cervical smear” OR TI “cervical screen” OR AB “cervical screen” OR TI “pap smear” OR AB “pap smear” OR TI  “pap test” OR AB “pap test” OR TI “papanicolaou smear” OR AB “papanicolaou smear” OR TI “papanicolaou test” OR AB “papanicolaou test” OR TI “mammography” OR AB “mammography” OR TI “prostate-specific antigen” OR AB “prostate-specific antigen” |
| #2 | TI “Web” OR AB “web” OR TI “Internet” OR AB “Internet” OR TI “Net surfing” OR AB “net surfing” OR TI “technology-based” OR AB “technology-based” OR TI “Information Technology” OR AB “information technology” OR TI “Website” OR AB “website” OR TI “information and communication technology” OR AB “information and communication technology” OR TI “eHealth” OR AB “eHealth” OR TI “telemedicine” OR AB “telemedicine” OR TI “ICT” OR AB “ICT” OR TI “Telehealth” OR AB “telehealth” OR TI “email” OR AB “email” OR MH “world wide web” OR MH “internet” OR MH “information technology” |
| #1 | TI “Implement*” OR AB “implement*” OR TI “Intervention” OR AB “intervention” OR  TI “adoption” OR AB “adoption” OR TI “Integration” OR AB “integration” OR TI Feasibility OR AB “feasibility” OR TI “Knowledge transfer” OR AB “knowledge transfer” OR  TI “Diffusion” OR AB “diffusion” OR TI “Disseminat*” OR AB “disseminat*” OR TI “Translat*” OR AB “translat*” OR TI “Quality” OR AB “quality” OR TI “Program” OR AB “program” OR TI “Innovation” OR AB “innovation” OR MH “program implementation” OR MH "health services accessibility" OR MH “health care delivery” OR MH “program evaluation” OR MH “health care reform” |

1. Web of Science

| No. | Search strategy |
| --- | --- |
| #4 | (#1 AND #2 AND #3) |
| #3 | TS=(Screening OR Health check* OR Health examination OR health promotion OR occult blood OR sigmoidoscopy OR colonoscopy OR cervical smear OR cervical screen OR pap smear OR pap test OR papanicolaou smear OR papanicolaou test OR mammography OR prostate-specific antigen) |
| #2 | TS=(Web OR Internet OR Net surfing OR technology-based OR Information Technology OR Website OR information and communication technology OR eHealth OR telemedicine  OR ICT OR Telehealth OR email) |
| #1 | TI=(Implement* OR Intervention OR Adoption OR Integration OR Feasibility OR Knowledge transfer OR Diffusion OR Disseminat* OR Translat* OR Quality OR Program OR Innovation) |

1. PsycINFO via Ovid

| No. | Search strategy |
| --- | --- |
| #4 | (#1 AND #2 AND #3) |
| #3 | Exp health screening/ or Exp screening tests/ or (Screening or Health check* or Health examination or health promotion or occult blood or sigmoidoscopy or colonoscopy or cervical smear or cervical screen or pap smear or pap test or papanicolaou smear or papanicolaou test or mammography or prostate-specific antigen).ab,ti. |
| #2 | exp internet/ or (Web or Internet or Net surfing or technology-based or Information Technology or Website or (information and communication technology) or eHealth or  telemedicine or ICT or Telehealth or email).ti,ab. |
| #1 | (Implement* or Intervention or Adoption or Integration or Feasibility or 'Knowledge transfer' or Diffusion or Disseminat* or Translat* or Quality or Program or Innovation).ti,ab. |

1. Embase via Ovid

| No. | Search strategy |
| --- | --- |
| #4 | (#1 AND #2 AND #3) |
| #3 | Exp health screening/ or Exp screening tests/ or (Screening or Health check* or Health examination or health promotion or occult blood or sigmoidoscopy or colonoscopy or cervical smear or cervical screen or pap smear or pap test or papanicolaou smear or papanicolaou test or mammography or prostate-specific antigen).ab,ti. |
| #2 | exp internet/ or (Web or Internet or Net surfing or technology-based or Information Technology or Website or (information and communication technology) or eHealth or  telemedicine or ICT or Telehealth or email).ti,ab. |
| #1 | (Implement* or Intervention or Adoption or Integration or Feasibility or 'Knowledge transfer' or Diffusion or Disseminat* or Translat* or Quality or Program or Innovation).ti,ab. |
